# The Hon. Sydney Holland on Differences of Opinion

**Published:** 1912-01-20

**Authors:** Sydney Holland

**Affiliations:** Chairman London Hospital. Whitechapel, E.


### THE HON. SYDNEY HOLLAND ON DIFFERENCES OF OPINION.

SIR,—You comment on the fact that the Secretary of the London Hospital and the Governors differ from me as to the probable effect on the subscriptions to the London Hospital from the passing of the Insurance Bill. That is not to be wondered at. I find myself nearly alone, Mr. Danvers Power agrees, in thinking that after a time when people grow used to the Act—if it ever gets into working order, which seems to me doubtful—they will not withdraw their subscriptions.

At the present moment I am having a bad time, and we are receiving a great number of letters refusing to renew subscriptions and refusing to help. But these refusals, so far, are from small subscribers. On the other hand, we have Sir Ernest Cassel sending a magnificent gift to hospitals; we at the London have had Christmas presents of £1,000 from one man, £300 from another, £100 each from two others, all quite unexpected. The balance so far is on my side. My reason for thinking that the effect will wear off is that as regards the employer the payment of 13s. per year a head for every employé is not a very serious extra tax and cannot be compared for one moment with other, and more serious, taxation which has not had the disastrous effect prophesied for it, *e.g.* Sir V. Harcourt's death duties and their subsequent increases.

As regards working-men's subscriptions, the best of them—*i.e.* those who were in Friendly Societies before—will certainly be better off after the Act is in working; and as regards the general public, they may need a little teaching, but will learn in time that the Act in no way gives "adequate" medical relief as promised by Mr. Lloyd George, but is only a help to people in trifling ailments. They will learn that in all serious illnesses the working-classes must have resort to the hospitals.

On the other hand, we ought to be relieved of about one-third of our out-patients, one-third of our out-patient drug bill, and we ought to receive payment for much of our work in the several directions indicated by the Act.

Perhaps I am too sanguine. You may feel well assured that I am not in a happy frame of mind. The London Hospital will have to pay about £800 a year for insurance for employés, and the tax on "reversions" (the tax on unearned increment, as some people call it) has already cost us £817.

Unless, therefore, people do help us more, or if the present general indignation becomes epidemic, I shall lose my bet with you that no beds will be closed.

The meeting I have called is not to protest against the Bill, but to settle upon a policy how to carry it out, especially as regards out-patients.—Yours faithfully,

Whitechapel, E., January 9. Sydney Holland, Chairman London Hospital.

